# Sika pilose antler type I collagen promotes BMSC differentiation via the ERK1/2 and p38-MAPK signal pathways

**DOI:** 10.1080/13880209.2017.1397177

**Published:** 2017-11-08

**Authors:** Yanshuang Wang, Su Luo, Dafang Zhang, Xiaobo Qu, Yinfeng Tan

**Affiliations:** a Center for New Medicine Research, Changchun University of Traditional Chinese Medicine, Changchun, China;; b School of Basic Medicine, Beihua University, Jilin, China;; c Jilin City People’s Hospital, Jilin, China

**Keywords:** Osteoblast, signal transduction pathways, molecular mechanisms

## Abstract

**Context:** Sika pilose antler type I collagen (SPC-I) have been reported to promote bone marrow mesenchymal stem cell (BMSC) proliferation and differentiation. However, the underlying mechanism is still unclear.

**Objective:** This study investigates the molecular mechanisms of SPC-I on the BMSC proliferation and differentiation of osteoblast (OB) *in vitro*.

**Material and methods:** The primary rat BMSC was cultured and exposed to SPC-I at different concentrations (2.5, 5.0 and 10.0 mg/mL) for 20 days. The effect of SPC-I on the differentiation of BMSCs was evaluated through detecting the activity of alkaline phosphatase (ALP), ALP staining, collagen I (Col-I) content, and calcified nodules. The markers of osteoblastic differentiation were evaluated using RT-PCR and Western-blot analysis.

**Results:** SPC-I treatment (2.5 mg/mL) significantly increased the proliferation of BMSCs (*p* < 0.01), whereas, SPC-I (5.0 and 10.0 mg/mL) significantly inhibited the proliferation of BMSCs (*p* < 0.01). SPC-I (2.5 mg/mL) significantly increased ALP activity and Col-I content (*p* < 0.01), and increased positive cells in ALP staining and the formation of calcified nodules. Additionally, the gene expression of ALP, Col-I, Osteocalcin (OC), Runx2, Osterix (Osx), ERK1/2, BMP2 and p38-MAPK, along with the protein expression of ERK1/2, p-ERK1/2, p-p38 MAPK were markedly increased in the SPC-I (5.0 mg/mL) treatment group (*p* < 0.01) compared to the control group.

**Discussion and conclusions:** SPC-I can induce BMSC differentiation into OBs and enhance the function of osteogenesis through ERK1/2 and p38-MAPK signal transduction pathways and regulating the gene expression of osteogenesis-specific transcription factors.

## Introduction

Osteoporosis, one of the most common bone diseases characterized by bone microstructure deterioration in addition to a reduction in bone mass, becomes a serious threat to post-menopausal women and elderly men (Lodie et al. 2002; Meier et al. 2005). As the aged population increases, the number of osteoporosis patients increases to an estimated patient population of 200 million worldwide. Long-term use of Western medical treatments for the effective prevention and control of osteoporosis is liable to result in a variety of side effects (Szulc et al. 2008). The development of traditional Chinese medicine (TCM) has become a research topic of interest, and many natural medicines have been reported for use in the treatment of osteoporosis (Li et al. 2003).

Historically, antler velvet, which is a young horn of male *Cervus nippon* Temminck or *Cervus elaphus* Linnaeus, is rare traditional animal-derived medicine. Traditional use of antler velvet as medicines has been extensively recognized. Theoretically, a concept that the kidney governs the bones had been recorded for several thousands of years and it remains popular in China. The deficiency in kidney function is deemed to be closely related to osteoporosis among bones and joints (Okazaki and Sandell 2004; Bauer et al. 2009). The velvet and its extracts are commonly administered to prevent and treat kidney deficiency related to osteoporosis in order to strengthen bone and muscles, reduce chronic joint pain (Wu et al. 2013). In addition, recent scientific investigations have documented that antler velvet preparations were able to promote chondrocyte and osteoblast proliferation and antibone resorption in adjuvant-induced arthritic rats (Kim et al. 2005). Therefore, the antler velvet’s biological and bioremdial properties need to be fully known and understood. Structurally, velvet antler components are the same as human bone having a calcium phosphate matrix (73%) with other organic material (23%). In organic material, the Sika pilose antler type I collagen (SPC-I) (80%) is mainly made of fibrous tissues.

In recent years, bone marrow mesenchymal stem cells (BMSCs) have become one of the most promising sources for precursor cells in tissue engineering (Takeda et al. 2001). BMSCs have strong proliferation and self-renewal ability and a potential for multi-directional differentiation. Research on single-direction differentiation of BMSCs to osteoblasts has great significance for anti-osteoporosis treatment (Center et al. 2011). Osteoclastogenesis formation needs a cell-to-cell interaction of hematopoietic precursor cells with osteoblasts. One of the key factors inducing the osteoblast is the receptor activator of nuclear factor-κB (RANK) and its ligand (RANKL), which belongs to the tumour necrosis factor family. RANK, as the receptor of RANKL, is involved in the activation of signal pathways such as mitogen-activated protein kinases (MAPKs), which include extracellular signal-regulated kinase (ERK) and p38. Similar to the MAPK pathway, the bone morphogenetic protein (BMP) signalling pathway also regulates the osteoblast differentiation and proliferation.

We have previously isolated the native SPC-I and analysed its amino acid sequence (Na et al. 2016). Moreover, we used BMSC as a model system and found that the SPC-I promoted the differentiation and proliferation of BMSC by activating the BMP-2/Smad signalling pathway. However, recent advances have suggested that multiple signalling pathways participate in the development of BMSC into osteoprogenitors, the current results were deficient with regards evidence of other pathways involved, which required the scrutiny of collagen-induced ERK1/2 and p38-MAPK signalling pathways.

In the present study, we proved the hypothesis that there was a cross-talk mechanism between ERK1/2 and p38-MAPK that SPC-I acted on the differentiation and proliferation of BMSC. We found that SPC-I promoted BMSC differentiation into osteoblast through ERK1/2 and p38-MAPK signal transduction pathways in addition to BMP-2/Smad signalling pathway. These results advocate SPC-I as a theoretical and experimental basis for the development of deer antler products.

## Material and methods

### Reagents

Dulbecco’s modified Eagle’s medium low glucose (DMEM-LG), fetal bovine serum (FBS), trypsin, dexamethasone (Dex), β-glycerophosphate (β-GP) and vitamin C (vit C) were purchased from Gibco (Grand Island, NY). U0126, antibodies specific for ERK1/2, phospho-ERK1/2, phospho-p38-MAPK monoclonal antibody, IgG/HRP and β-actin polyclonal antibody were purchased from Cell Signaling Technology (Beverly, MA). Gomori staining kit, alizarin red dye solution, rat collagen-I (Col-I) and ELISA detection kit were obtained from the Jiancheng Bioengineering Institute (Nanjing, China). TRIzol was purchased from Invitrogen (Carlsbad, CA). Primer synthesis and RT, PCR kits were purchased from Sangon Biotech (Shanghai, China). The CCK-8 kit and Western blotting reagents were purchased from Beyotime Biotechnology Institute (Haimen, China).

### Isolation and culture of BMSC

Rat BMSCs were isolated and confirmed using a modification of methods previously described (Na et al. 2016) and put in the freezer (−80 °C) for further use. The study was made to comply with the Animal Research: Reporting In Vivo Experiments (ARRIVE) guidelines (Changchun University of Chinese Medicine ethical approval number: Protocol Number 2014-08-15). The obtained BMSCs recruited and expanded for the following experiments. Briefly, we removed cryopreserved BMSC from the freezer, put the cryopreserved tubes in a water bath 37 °C, shook to melt, and after disinfection and filtration, removed the cell suspension by suction, and injected it into a centrifuge tube, added DMEM-LG complete medium 5 mL, centrifuge for 5 min at 1500 rpm, and discarded the supernatant. Then, we added 5 mL DMEM-LG complete medium, containing 20% FBS, 100 U/mL of penicillin, and 100 U/mL of streptomycin, inoculated 25 cm^2^ culture bottles at 37 °C, under 5% CO_2_ and a saturated (100% relative humidity) atmosphere for incubation. The medium was changed for fresh medium every 3 days. After waiting for cell growth of 80–90%, according to 1:2 proportions for sub-culture, all cells were passaged every week, and the third or fifth passages of cells were used in the study. After the third passage, cells were collected and seeded on six-well plastic plates at an initial density of 5 × 10^4^ cells/well.

### Preparation of SPC-I

SPC-I was extracted by a trypsin hydrolysis method as described as previously (Na et al. 2016), and protein concentration was 56.89%. To exclude the other protein peptide residues, high performance liquid chromatography (Agilent 1100 series; Agilent Technologies, Lexington, MA) was used to purify the SPC-I. Eluents were 97% aqueous acetonitrile containing 1.0 mol/L sodium acetate (pH 6.5) and 80% aqueous acetonitrile. The total of SPC-I in different molecular sizes distribution with HPLC presented 9 and 15 kDa with 96.89% purity, which contained seven essential amino acids, and glycine is the most dominant amino acid (32.1%). Before application, the bacterial endotoxin test was performed by LAL Chromogenic Endpoint assay (Hycult Biotech, Uden, Netherlands) to testify that the SPC-I was free of endotoxin, and then the SPC-I was dissolved in deionized water at concentration of 2.5, 5.0, 10.0, 20.0, and 40.0 mg/mL.

### BMSC proliferation assay

BMSCs proliferation was detected using cell counting kit-8 (CCK-8) according to the manufacturer’s instructions. Briefly, BMSCs at passage three were seeded into 96-well plates at a density of 2.5 × 10^4^ cells/mL and allowed to grow for 24 h. The cultured cells were divided randomly into three groups: a control group (blank control), an SCP-I group, and a Dex group (positive control). In the Dex group, BMSCs were grown in medium containing Dex at 10^−8^ M. In the control group, cells were cultured in serum-free culture medium. In the SPC-I group, cells were cultured with additional SPC-I at distinct concentrations, i.e., 2.5, 5.0, 10.0, 20.0, and 40.0 mg/mL. The medium was refreshed every 3 days and supplemented with SPC-I at a different concentration. At the end of the treatment, 20.0 μL of a 5.0 mg/mL CCK-8 was added to each well. After 1 h of incubation at 37 °C, the supernatant was collected and the optical density (OD) was determined using an absorbance microplate reader (Bio Tex, Winooski, VT) set at a wavelength of 450 nm. The growth curve of the groups was drawn. Three independent repeats were performed for all experiments.

### Measurement of several markers of osteoblastic differentiation

The BMSCs at passage two were seeded into 24-well plastic plates in 1 mL medium, at a density of 2.5 × 10^4^ cells/mL. The cultured BMSCs were divided randomly into three groups: a control group, an SCP-I group, and a Dex group. In the Dex group, BMSCs were grown in medium with Dex at 10^−8^ M. In the control group, cells were grown in medium with 10 mM β-GP and 50.0 µg/mL ascorbic acid. In the SPC-I group, cells were cultured with additional SPC-I at different concentrations (2.5, 5.0, and 10.0 mg/mL) in the presence of 10 mM β-GP in addition to 50.0 µg/mL ascorbic acid. After 3 days, the culture medium was changed and supplemented with drugs according to different groups. The culture medium was altered every 3 days. Next, the supernatant was collected on the 6^th^, 9^th^, and 12^th^ days, respectively, and several markers of osteoblastic differentiation were evaluated every 4 days until the 20^th^ day.

The intracellular type I collagen was analysed using an ELISA kit (C0017#, Haimen, China) according to the manufacturer’s instructions. In brief, the supernatant was fixed in rat collagen I monoclonal antibody in a microtitre plate in advance and incubated with biotin-labelled anti-collagen I antibodies and streptavidin-labelled horseradish peroxidase (HRP)-conjugated antibodies combined with an immune complex. At the end of incubation, the substrate was added, and the colour development therein was quantified using a spectrophotometer (Bio Rad, Hercules, CA) at 450 nm. From the linear regression equation fitted to the standard curve, the corresponding sample concentration was calculated.

Intracellular alkaline phosphatase (ALP) activity was detected by a spectrophotometric method. The BMSCs were seeded into 24-well plastic plates in 1 mL medium, at a density of 2.5 × 10^4^ cells/mL. The complete medium was replaced with conditioned medium after 24 h, and the latter was altered every 3 days. Next, the supernatant was collected on the 6^th^, 9^th^, and 12^th^, days, respectively, and ALP activity was detected using an ALP reagent kit according to the manufacturer’s instructions. The matrix was added to 100 mL supernatant for 15 min at 37 °C, producing 1 mg phenol as a Kim unit. The results were transformed into international units (1 Kim units =7.14 international units u/L).

During the BMSCs in osteogenic differentiation condition, the cells were stained using an improved Gomori method and alizarin red staining as described previously (Prophet et al. 1994) on the 9^th^ and 14^th^ days, respectively. The cells were washed twice with PBS and set in 95% ethanol for 10 min. Next, for an improved Gomori method, β-glycerin sodium phosphate was added and incubated at 37 °C for 4 h, and then added to 2% cobalt nitrate for 5 min and to ammonium sulphide for 2 min. For alizarin red staining, 0.1% alizarin red was in addition to the cells for 30 min. ALP-positive cells and calcium nodules were observed and their images acquired using an optical microscope.

### RNA isolation and analysis of the mRNA expression of ALP, Col-I, OC, RunX2, and Osx

The BMSCs at passage three were seeded into six-well plastic plates in 1 mL medium, at a density of 2.5 × 10^4^ cells/mL and divided randomly into three groups as described for the BMSC differentiation assay. The culture medium was altered every 3 days and was cultured until the 9^th^ day. The cell pellets were collected and total RNA was extracted with TRIzol reagent. cDNA was synthesized with a cDNA synthesis kit according to the manufacturer’s instructions. The relative amounts of each gene mRNA expression to G3PDH were measured by quantitative reverse transcripts PCR with the SYBR premixed system and the specific primer sequences for ALP, Col-I, OC, RunX2, and Osx in addition to ERK1/2, BMP2, and p38-MAPK as described in the protocol (Lee et al. 2000; Na et al. 2016). qRT-PCR was performed using the standard kit. The relative transcript levels of the target gene were normalized to that of G3PDH using the 2^−ΔΔCt^ assay.

### Evaluation of the mRNA expression of ERK1/2, BMP2 and p38-MAPK

The BMSCs at passage three were seeded into six-well plastic plates in 1 mL medium, at a density of 2.5 × 10^4^ cells/mL and divided randomly into four groups, three out of four groups were as described in the BMSC differentiation assay and the inhibitory group was supplemented with U0126. The culture medium was altered every 3 days and was cultured until the 9^th^ day. Except for the control and inhibitory groups, others were supplemented with culture medium at the same volume of U0126 to the terminal concentration of 50 μM and incubated for 2 h before changing the medium. The protocol of extracting the absolute RNA, cDNA synthesis, qRT-PCR, and evaluation of results was as described above.

### Western-blot analysis of the proteins of ERK1/2, p-ERK1/2 and p-p38-MAPK

The BMSCs at passage three were seeded into six-well plastic plates in 1 mL medium, at a density of 2.5 × 10^4^ cells/mL and were divided into groups as described above in addition to protocol in addition of U0126 and changing of the medium. The culture medium was altered every 3 days and was cultured until the 9^th^ day. The cell pellets were collected and lysed with IP lysis buffer (Thermo Fisher Scientific, Waltham, MA), at the same time the cell lysates were heat-denatured for 5 min. The protein from each sample was isolated by sodium dodecyl sulphate–polyacrylamide gel electrophoresis (SDS–PAGE) on a 12% separating gel and a 5% stacking gel. They were then transferred onto a nitrocellulose membrane and incubated overnight at 4 °C, and 30 V. The proteins were detected using rabbit anti-rat ERK1/2 (1:200), p-ERK1/2 (1:200), and p-p38-MAPK (1:200) antibodies as the primary antibodies and HRP-conjugated goat anti-rabbit IgG (1:500) as the secondary antibody. Anti-β-actin was used as an internal protein-loading control. The membranes were detected by a DAB Horseradish Peroxidase Colour development kit (Haimen, China). The protein bands were visualized and analysed using ChemiDoc image J software (Bio-Rad Laboratories Inc., Hercules, CA).

### Statistical analysis

All groups were established in duplicate. The datum was expressed as the mean ± standard deviation (SD), calculated from at least three independent experiments. Statistical comparisons were done using the two-tailed Student’s *t*-test. The analysis was carried out using SPSS16.0 (SPSS Inc., Chicago, IL) software package. Statistical significance was designated as *p* < 0.05.

## Results

### Effect of SPC-I on proliferation of BMSCs

**Figure 1.**
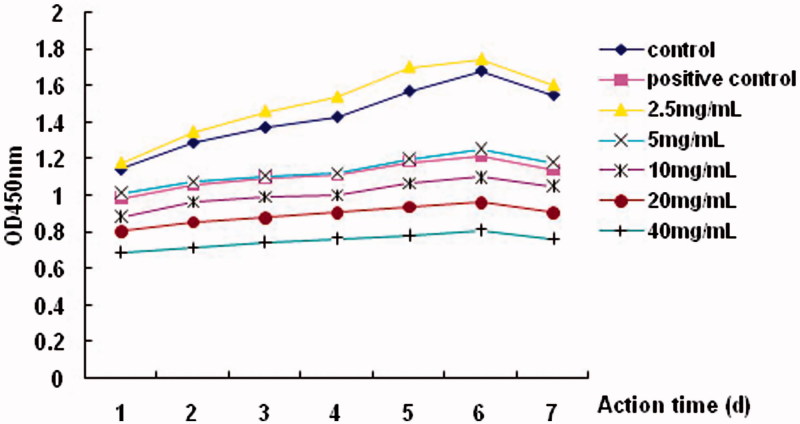
Assessment of SPC-I on cell proliferation of BMSC via the CCK-8 assay.

The addition of different concentrations of SPC-I had different effects on the proliferation of BMSCs. Compared to the control group, the addition of 2.5 mg/mL of SPC-I significantly increased the OD values in a time- and dose-dependent manner; however, addition of 5.0–40.0 mg/mL inhibited proliferation (*p* < 0.01), except that 5.0 mg/mL of SPC-I had a proliferative effect on BMSC, which was similar to that in the positive control with Dex (*p* > 0.01) (Figure 1).

### SPC-I increased the Col-I expression, ALP activity,ALP-positive cells, and amount of calcium nodules

**Figure 2.**
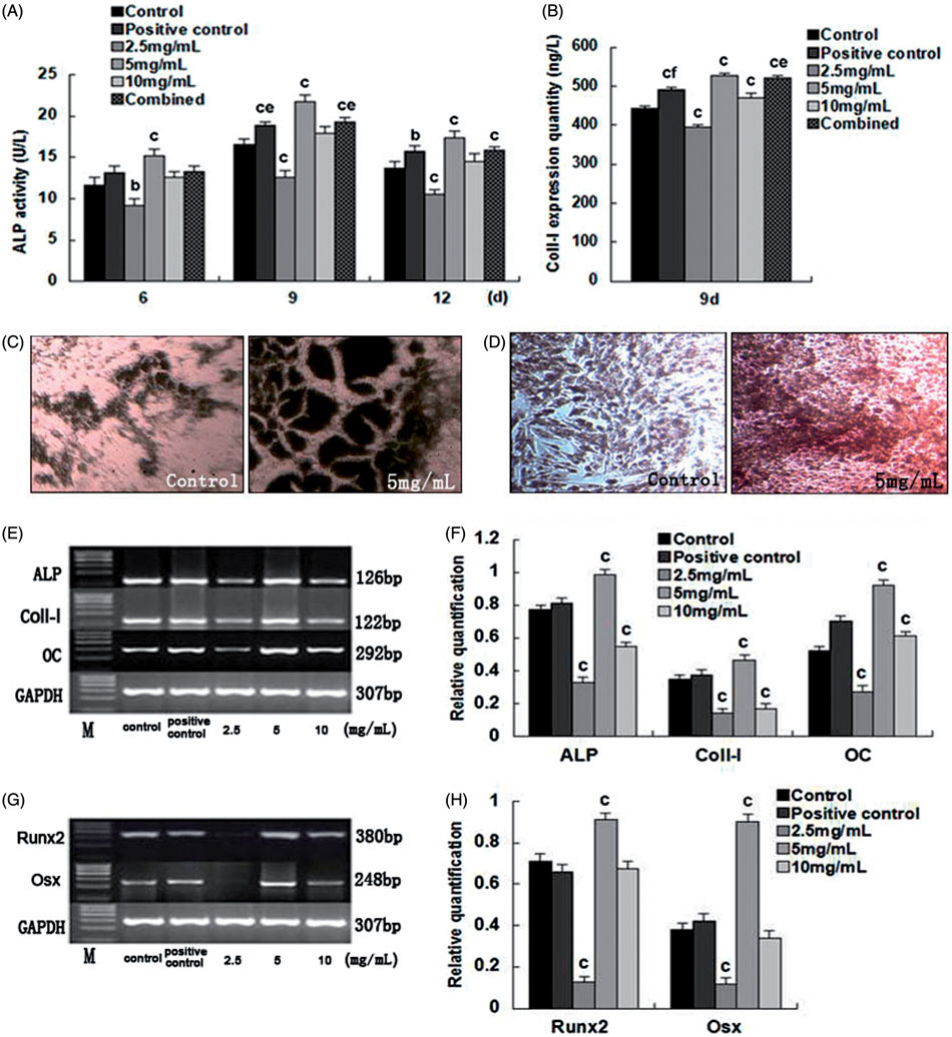
The effects and evaluation of SPC-I on BMSC differentiation into osteoblasts.

ALP activities were measured, and were highest on the 9^th^ day. The activity was lower when treated with 2.5 mg/mL SPC-I (*p* < 0.01); however, the activity was higher when treated with 5.0 mg/mL SPC-I (*p* < 0.05 *v*. the positive control and combined groups, *p* < 0.01 *v*. the control group). Similarly, ALP activity for the 10.0 mg/mL SPC-I group was high, but lower than that of the 5.0 mg/mL group (Figure 2(A)).

The 2.5 mg/mL SPC-I group showed a lower Col-I expression (*p* < 0.01 *v*. the control group); however, 5.0 mg/mL SPC-I elevated the Col-I expression (*p* < 0.05 *v*. the combined control; *p* < 0.01 *v*. the control and positive control groups). Similarly, the Col-I expression in the 10.0 mg/mL group increased (*p* < 0.01), but was less than that in the 5 mg/mL group (Figure 2(B)).

The cytoplasm of ALP-positive cells showed light gray, gray, and black, deep black particles or large flake black precipitates, using the improved Gomori method. The ALP-positive cell counts increased considerably in the 5.0 mg/mL SPC-I group (Figure 2(C)). The cell monolayers overlapped, clustered, and formed a dense tissue, due to deformation caused by cell shrinkage. The tissue was incorporated within a secreted matrix, subjected to calcium salt deposition, matrix mineralization, and thus formed opaque areas. Red and radial calcium nodules were noted with the alizarin red staining. SPC-I (5.0 mg/mL) markedly promoted the formation of calcified nodules (Figure 2(D)).

### SPC-I increased gene expression during BMSC differentiation into osteoblasts

The RT-PCR indicated consistently amplified bands of ALP, Col-I, OC, Runx2, and Osx. The gene expression of ALP, Col-I, OC, Runx 2, and Osx was lowest in the 2.5 mg/mL SPC-I group (*p* < 0.01) and highest in the 5.0 mg/mL SPC-I group (*p* < 0.01); however, the expression of the 10.0 mg/mL SPC-I group was unclear (Figure 2(E–H)).

**Figure 3.**
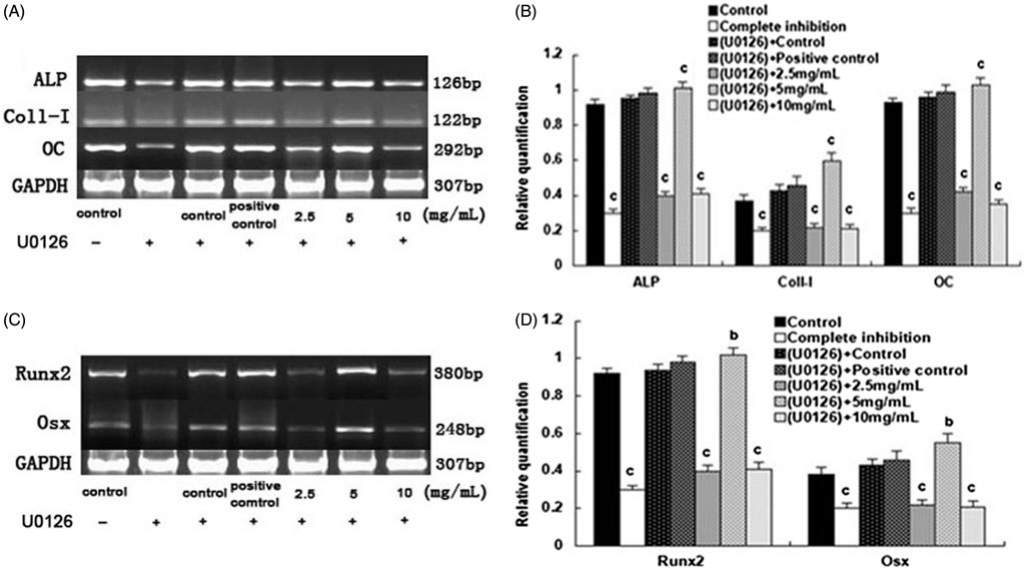
The effect of SPC-I on the gene expression of osteogenesis markers and gene and osteogenesis transcription regulation gene (with U0126).

**Figure 4.**
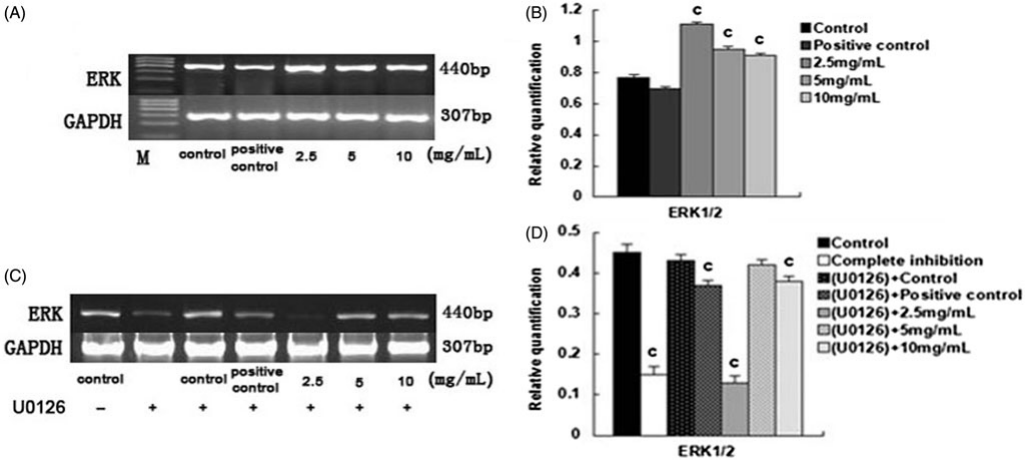
The effect of SPC-I on the gene expression of ERK1/2 during BMSC differentiation into osteoblasts.

**Figure 5.**
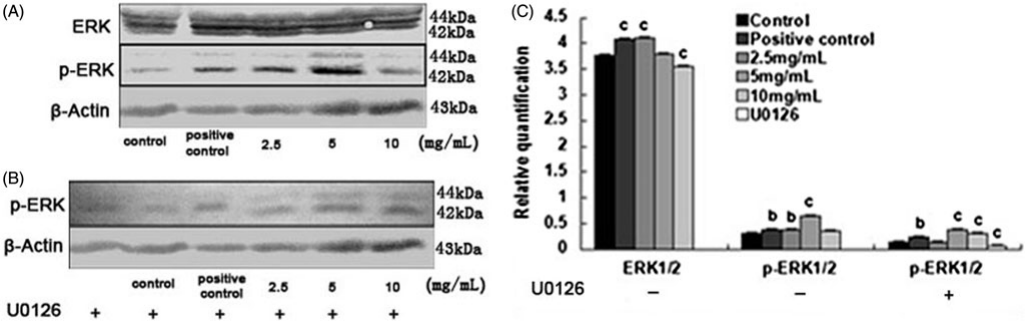
The effect of SPC-I on the protein expression of ERK1/2 and p-ERK1/2 (without and with U0126).

Upon the addition of U0126, the gene expression of ALP, CoI-I, OC, Runx2, and Osx in the 2.5 mg/mL and the 10.0 mg/mL SPC-I group, as well as complete inhibition groups, decreased (*p* < 0.01); however, the gene expression was elevated in the SPC-I 5 mg/mL SPC-I group (*p* < 0.01) (Figure 3(A–D)). Simultaneously, effect of SPC-I on the gene expression of ERK1/2 (p42/44-MAPK) during BMSC differentiation into osteoblasts exhibited the same phenotype. The gene expression of ERK1/2 was elevated in the 2.5, 5.0, and 10.0 mg/mL SPC-I groups; the 2.5 mg/mL SPC-I group showed the highest expression (*p* *<* 0.01) without U0126 (Figure 4(A,B)). The gene expression of ERK1/2 was reduced in the 2.5 and 10.0 mg/mL SPC-I groups and in the complete inhibition and positive control groups. The 2.5 mg/mL SPC-I group had a lower expression (*p* < 0.01) with U0126; however, the expression in the 5.0 mg/mL SPC-I group was not reduced significantly (*p* > 0.05) (Figure 4(C,D)). Without U0126, the gene expression of BMP2 was elevated in the SPC-I 5 mg/mL and positive control groups, the SPC-I 5.0 mg/mL group was highest (*p* < 0.01); however, the BMP2 expression in the 2.5 and 10 mg/mL SPC-I groups decreased. Both in the absence, and presence, of U0126, the gene expression of p38-MAPK was highest in the 5 mg/mL SPC-I group (*p* < 0.01) and that of the 2.5 and 10.0 mg/mL SPC-I groups decreased (*p* < 0.01) (Figure 5(A,C)); however, the gene expression of p38-MAPK was much increased after treatment with U0126 (*p* < 0.01) with the exception of the 2.5 mg/mL group (Figure 5(B,C)).

### Effect of SPC-I on the protein expression of ERK1/2, p-ERK1/2, and p-p38-MAPK

**Figure 6.**
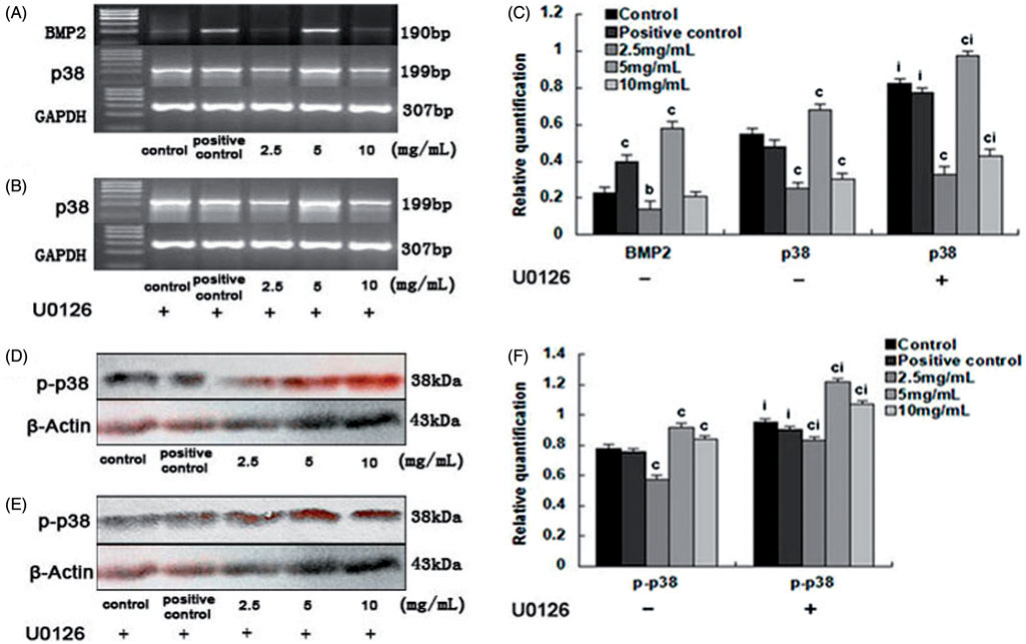
The effect of SPC-I on the expression of BMP2 and p38-MAPK.

Without U0126, the protein expression of ERK1/2 was elevated in the 2.5 mg/mL SPC-I and positive control groups and decreased in the 10.0 mg/mL SPC-I group (*p* < 0.01); however, it was not significantly decreased in the 5.0 mg/mL SPC-I group (*p* > 0.05). The protein expression of p-ERK1/2 was elevated in the 2.5 and 5.0 mg/mL SPC-I groups and in the positive control groups. The expression in the 5.0 mg/mL SPC-I group was the highest (*p* < 0.01). It was not decreased significantly in the 10.0 mg/mL SPC-I group (Figure 6(A,C)).

With U0126, the protein expression of p-ERK1/2 was promoted in the 5.0 and 10.0 mg/mL SPC-I groups and in the positive control groups and that in the 5.0 mg/mL SPC-I group was the highest (*p* < 0.01). It was reduced in the 2.5 mg/mL SPC-I and the complete inhibition groups (Figure 6(B,C)).

Both in the absence, and presence, of U0126, the protein expression of p-p38-MAPK was elevated in the 5.0 and 10.0 mg/mL SPC-I groups. The 5.0 mg/mL SPC-I group was highest (*p* < 0.01). In contrast, the expression in the SPC-I 2.5 mg/mL and the positive control groups decreased (Figure 6(D,F)); however, the protein expression of p-p38-MAPK was significantly increased after treatment with U0126 (*p* < 0.01) (Figure 6(E,F)).

## Discussion

BMSCs are pluripotent stem cells that have the potential for multi-directional differentiation into bone, cartilage and many other cell types. Several studies *in vivo* and *in vitro* have proved that osteogenic capacity and differentiation of BMSCs into these targets lineages require the influence of local stimuli (Sharp and Magnusson 2008). When BMSCs were grown *in vitro*, only some part of the cells can automatically differentiate into osteoblasts. Differentiation largely depends on differentiation inducers, which include β-GP, Dex, and vit C (Wenstrup et al. 1996).

In this study, we selected β-GP + vit C as the negative control group, β-GP + vit C + Dex as the positive control group, and β-GP + vit C + SPC-I (minimal, medium, and high doses) as the experimental group. The inhibitory effect of 5.0 mg/mL SPC-I of BMSC proliferation is similar to that of the positive control of Dex. Thus, 2.5, 5.0, and 10.0 mg/mL were selected for subsequent studies based on the 50% effective concentration.

The osteogenetic differentiation of uncommitted BMSCs is an orderly, fundamental process, which is often accompanied by the activation and deactivation of some restricted transcription factors (Leslie et al. 2012). A better understanding the mechanism during BSCs osteogensis is essential for development of activators or repressors. ALP is the earliest osteogenetic marker for BMSC differentiation into osteoblasts. ALP activity is regarded as an indicator from which BMSCs differentiate into osteoblasts and for the functional state of osteoblasts. Additionally, the Col-I content is an indicator of the maturity of osteoblasts. It has been documented that the Col-I metabolism indices accurately reflect bone metabolism with higher sensitivity, specificity, and stability than the bone biochemical indices employed in the past (Dawson-Hughes et al. 2012). At the meantime, osteocalcin (OC) is a mature marker of osteoblasts and is considered to be the most common characteristic of the differentiation of BMSCs to osteoblast markers. Alizarin red specifically stains calcium deposits and can directly reflect osteoblast mineralization, which is required to form calcified osseous tissue eventually (Kim et al. 2009).

Runx2/Cbfa1 is a distinctive transcriptional regulation factor in the process of osteoblast differentiation, which plays a key role and is referred to as the regulation gene; it was identified as osteogenesis-specific transcription activating factor. It can govern the expression of many osteogenetic markers. Osx is specifically expressed in the development of bone tissue and is a key molecule in osteogenetic differentiation and bone formation. It was noted that during BMSC differentiation into osteoblasts, Runx2/Cbfa1 was expressed first, followed by the opening of the cell differentiation ‘switch’: this process causes the BMSCs to differentiate only into osteoblasts or cartilage cells. Osx is downstream of Runx2; the expression of Osx is regulated by Runx2, and the major function of Osx is to promote stem cell differentiation to osteoblasts and to inhibit stem cell differentiation into cartilage cells (Whitehurst et al. 2004; Boonen et al. 2007).

In the course of BMSCs osteogenesis, our findings showed that the 5 mg/mL SPC-I up-regulated the mRNA expression of Runx2, thereby triggered the ‘molecular switch’, including by further elevating the mRNA expression of Osx, which promoted the expression of marker gene for osteoblast differentiation, such as ALP, Col-I and OC, improving Col-I expression and ALP activity, all of which may contribute to promote osteoblast differentiation, maturation, and bone formation. Interestingly, we found that SPC-I affects BMSC proliferation and differentiation in a time- and dose-dependent manner. However, the proliferative effect was attenuated at 5.0 mg/mL SPC-I as time increased compared with the effect at 2.5 mg/ML. Similar effects have been documented for Dex treatment. Both high concentrations of Dex and SPC-I would reduce the proliferation capacity and induce osteoblast commitment and maturation. The mechanisms by which the different concentrations of SPC-I activate and suppress the osteoblast differentiation of BMSCs are likely to be partially involved (receptors/BMP-2/Smad signalling pathway or ERK/MAPK signalling pathway).

All of the available evidence also supported a role for the ERK/MAPK pathway in osteoblast differentiation (Lee et al. 2000; Sun et al. 2006). ERK1/2 is a protein kinase with a relative molecular weight of 44 kD/42 kD and has a homology of 90%. In non-stimulated cells, ERK1/2 is in its non-phosphorylated state. Upstream molecular phosphorylation control signal MAPK kinase (MAPK kinase, MAPKK) activates the phosphorylated form of ERK1/2: p-ERK1/2. The expression of p-ERK1/2 can reflect the activation level of the ERK1/2 channel. Generally, when Smad, a specific transcription factor, is phosphorylated by a BMP receptor, BMP signalling is activated and MAPK is also phosphorylated. The activation of MAPK results in the cytoplasmic retention of Smad and a subsequent translocation to the nucleus. It can also phosphorylate other enzymes, change their activity, and regulate the gene expression. U0126 is a specific, and efficient, inhibitor of MEK1/2: it blocks ERK1/2 phosphorylation through the direct inhibition of MEK1/2 activity. It can prevent MEK1/2 nuclear transfer activation to produce a singular biological effect; however, other molecules in the MAPK pathway (JNK/SAPK, p38 MAPK, etc.) do not have a blocking effect.

To further investigate the association between p-ERK1/2 and ERK1/2, we detected whether SPC-I regulated the BMSCs osteoblast differentiation through p-ERK1/2 and ERK1/2 signalling pathway during osteogenesis. The RT-PCR study showed that the expression level of ERK1/2 was unique among the groups, suggesting that the signalling pathways of ERK1/2 were abnormally activated before the use of U0126. The mRNA expression levels activated the ERK1/2 signalling pathway, which is involved in SPC-I-induced BMSC differentiation of osteoblasts. The Western-blot results were in agreement with those of RT-PCR; the 2.5 mg/mL SPC-I group showed the highest expression of inactive ERK1/2 protein. Our preliminary studies showed that the 2.5 mg/mL group had the ability to promote BMSC differentiation to adipocytes. BMSCs have multi-directional differentiation potential, the adipogenesis and the osteogenesis occur in opposite directions, both trading off and taking turns; however, the protein expression of p-ERK1/2 was the highest in the 5.0 mg/mL SPC-I group, which showed that 5.0 mg/mL SPC-1 induced the ERK1/2 pathway to abnormally activate BMSC differentiation to osteoblasts, significantly improving the activation level of the ERK1/2 pathway, activating p-ERK1/2, and decreasing the cytoplasmic protein affinity function, and improving the transport of ERK1/2 into the nucleus and the transfer of extracellular information. Therefore, it regulated the downstream gene expression of osteoblast-specific transcription factors RunX2 and Osx, inducing BMSC differentiation to osteoblasts and further promoting the gene expression of marker genes of osteoblasts, such as ALP, Col-I, and OC. However, the overall expression of p-ERK1/2 was not high, so the activity of activated p-ERK1/2 itself may not be significant.

With regard to U0126, an ERK1/2-specific inhibitor, ERK1/2 protein phosphorylation was inhibited, and p-ERK1/2 protein production was decreased; however, ERK1/2 dephosphorylation and cytoplasmic protein production increased. Therefore, the level of activity along the activated ERK1/2 pathway was reduced, and the gene expression of specific transcription factors RunX2, Osx, and osteogenesis markers ALP, Col-I, and OC decreased. However, their expressions were not significantly decreased. The study showed that the activation of this pathway was not a case of ‘all or nothing’ after stimulation, but was the accumulated effect of stimulus intensity and time (Canalis et al. 2003; Aversa et al. 2013). Transiently activated ERK (activity was low) and consistently activated ERK (activity was high) due to differences in ‘into the nucleus’ stages, meant that the cells expressed different qualities, or quantities, of products, to produce a different effect in cell biological terms. With the most downstream of the MAPK signal transduction pathways, the strength of ERK1/2 signal transduction function not only depends on the activated form of p-ERK1/2, but is also closely associated with p-ERK1/2 activities. This experiment conjectures that, in the process of BMSC differentiation of osteoblasts, ERK1/2 signal transduction pathways were activated but may not have been the only pathway; there could be other signal transduction pathways involved.

BMPs are members of the transforming growth factor (TGF) family, and TGF-β is a strong factor in the promotion of stem cell osteogenetic differentiation (Maeda et al. 2004; Kalaszczynska et al. 2013). There are two less-BMP2-activated signal transduction pathways: the Smad pathway and the MAPK pathway. When the complex of BMP2, BMPR-II and BMPR-I forms on the cell surface, the activated BMPR-II phosphorylates the GS area of BMPR and activates the BMPR-I effect on the downstream Smads. When BMP2 binds with high affinity to BMPR-I forming a complex at the cell surface, BMPR-II is released into the cytoplasm. This occurs through BMPR-I forming different BISC (BMP2-induced signalling complex) polymers and through the indirect connection of the bridge proteins XIAP and TAB1 with TAK1 and TAK1 activation of p38-MAPK and transduction BMP signalling pathways. The p38 inhibitors reduced the phosphorylation of p38 with a simultaneous increase in ERK phosphorylation (Hotokezaka et al. 2002). MEK inhibitors reduce the phosphorylation of ERK and increase the extent of phosphorylation of p38.

In the present study, the 5.0 mg/mL SPC-I treatment significantly increased the BMP2 mRNA and p38-MAPK mRNA expression without U0126, and further significantly increased p38-MAPK mRNA and p-p38 MAPK protein expression with U0126. This demonstrated that the p38-MAPK pathway was activated when using U0126. It was speculated that the U0126 reduced ERK protein phosphorylation while the p38-MAPK protein phosphorylation levels increased simultaneously; thus, the ERK1/2 signal transduction pathway was blocked after using an ERK pathway inhibitor. Simultaneously, p-p38-MAPK activity was increased, further inducing the BMP2-p38-MAPK pathway. p-p38-MAPK directly phosphorylated the osteogenesis transcription factors Runx2 and Osx and indirectly activated some kinases and phosphorylated the downstream gene to express osteogenesis-specific marker genes, such as ALP, osteocalcin, and bone sialoprotein (Hassel et al. 2003; Hu et al. 2003). This result showed that SPC-I activated the ERK1/2 and p38-MAPK pathways, while regulating BMSC differentiation to osteoblasts. Additionally, ERK1/2 and p38-MAPK pathways were associated with each other, although the p38-MAPK pathway may have played the more prominent role. Interestingly, an additional finding concerns the p38 inhibitors activity. p38 inhibition with SB203580 (Sigma, St. Louis, MO), we did not find any increase or decrease of phosphorylated p38-MAPK (data not shown). Future experiments are need to clarify p38 inhibitors on the p38-MAPK pathway and to elucidate its regulatory mechanisms.

To summarize, this study suggested that SPC-I can induce BMSC differentiation into osteoblasts through the two signal transduction pathways—ERK1/2 and p38-MAPK—and can regulate the gene expression of Runx2 and Osx, which were specific transcription factors in this case. Our results have practical significance in further expounding molecular mechanisms of osteoporosis pathogenesis and are helpful for discovering novel molecular targets for osteoporosis through drug screening.

## References

[CIT0001] Aversa A, Bruzziches R, Song Y. 2013 *In vitro* proliferation and osteogenic differentiation of mesenchymal stem cells on nanoporous alumina. Int J Nanomed. 8:2745–2756. 10.2147/IJN.S44885 PMC3735283 23935364

[CIT0002] Bauer DC, Garnero P, Harrison SL, Cauley JA, Eastell R, Ensrud KE, Orwoll E; Osteoporotic Fractures in Men (MrOS) Research Group 2009 Biochemical markers of bone turnover, hip bone loss, and fracture in older men: the MrOS study. J Bone Miner Res. 24:2032–2038. 19453262 10.1359/JBMR.090526 PMC2791517

[CIT0003] Boonen S, Lips P, Bouillon R, Bischoff-Ferrari HA, Vanderschueren D, Haentjens P. 2007 Need for additional calcium to reduce the risk of hip fracture with vitamin D supplementation: evidence from a comparative metaanalysis of randomized controlled trials. J Clin Endocrinol Metab. 92:1415–1423. 17264183 10.1210/jc.2006-1404

[CIT0004] Canalis E, Economides AN, Gazzero E. 2003 Bone morphogenetic proteins, their antagonists, and the skeleton. Endocr Rev. 24:218–235. 12700180 10.1210/er.2002-0023

[CIT0005] Center JR, Bliuc D, Nguyen ND, Nguyen TV, Eisman JA. 2011 Osteoporosis medication and reduced mortality risk in elderly women and men. J Clin Endocrinol Metab. 96:1006–1014. 21289270 10.1210/jc.2010-2730

[CIT0007] Dawson-Hughes B, Looker AC, Tosteson AN, Johansson H, Kanis JA, Melton LJ. 2012 The potential impact of the national osteoporosis foundation guidance on treatment eligibility in the USA: an update in NHANES 2005–2008. Osteoporos Int. 23:811–820. 21717247 10.1007/s00198-011-1694-y

[CIT0008] Hassel S, Schmitt S, Hartung A, Roth M, Nohe A, Petersen N, Ehrlich M, Henis YI, Sebald W, Knaus P. 2003 Initiation of Smad-dependent and Smad-independent signaling via distinct BMP-receptor complexes. J Bone Joint Surg Am. 85-A(Suppl. 3):44–51. 10.2106/00004623-200300003-00009 12925609

[CIT0009] Hotokezaka H, Sakai E, Kananka K, Saito K, Matsuo K, Kitaura H, Yoshida N, Nakayama K. 2002 U0126 and PD98059, specific inhibitors of MEK, accelerate differentiation of RAW264.7 cells into osteoclast-like cells. J Biol Chem. 277:47366–47372. 12237315 10.1074/jbc.M208284200

[CIT0010] Hu Y, Chan E, Wang SX, Li B. 2003 Activation of p-38mitogen-activated protein kinase is required for osteoblast differentiation. Endocrin. 144:2068–2074. 10.1210/en.2002-220863 12697715

[CIT0011] Kalaszczynska S, Ruminski A, Platek E, Bissenik I, Zakrzewski P, Noszczyk M, Lewandowska-Szumiel M. 2013 Substantial differences between human and ovine mesenchymal stem cells in response to osteogenic media: how to explain and how to manage? Biores Open Access. 2:356–363. 24083091 10.1089/biores.2013.0029 PMC3776620

[CIT0012] Kamakura S, Moriguchi T, Nishida E. 1999 Activation of the protein kinase ERK5/BMK1 by receptor tyrosine kinases. Identification and characterization of a signaling pathway to the nucleus. J Biol Chem. 274:26563–26571. 10473620 10.1074/jbc.274.37.26563

[CIT0013] Kim HK, Kim JH, Abbas AA, Yoon TR. 2009 Alendronate enhances osteogenic differentiation of bone marrow stromal cells: a preliminary study. Clin Orthop Relat Res. 467:3121–3128. 18665432 10.1007/s11999-008-0409-y PMC2772902

[CIT0014] Kim KH, Kim KS, Choi BJ, Chung KH, Chang YC, Lee SD, Park KK, Kim HM, Kim CH. 2005 Anti-bone resorption activity of deer antler aqua-acupunture, the pilose antler of Cervus korean TEMMINCK var. mantchuricus Swinhoe (Nokyong) in adjuvant-induced arthritic rats. J Ethnopharmacol. 96:497–506. 15619570 10.1016/j.jep.2004.09.039

[CIT0015] Lee KS, Kim HJ, Li QL, Chi XZ, Ueta C, Komori T, Wozney JM, Kim EG, Choi JY, et al 2000 Runx2 is a commn target of transforming growth factor β1 and bone morphogenetic protein2, and cooperation between Runx2 and Smad5 induces osteoblast-specific gene expression in the pluri potent mesenchymal precursor cell line C2C12. Mol Cell Biol. 20:8783–8792. 11073979 10.1128/mcb.20.23.8783-8792.2000 PMC86511

[CIT0016] Leslie WD, LaBine L, Klassen P, Dreilich D, Caetano PA. 2012 Closing the gap in postfracture care at the population level: a randomized controlled trial. CMAJ. 184:290–296. 22184366 10.1503/cmaj.111158 PMC3281153

[CIT0017] Lodie TA, Blickarz CE, Devarakonda TJ, He C, Dash AB, Clarke J, Gleneck K, Shihabuddin L, Tubo R. 2002 Systematic analysis of reportedly distinct populations of multipotent bone marrow-derived stem cells reveals a lack of distinction. Tissue Eng. 8:739–751. 12459053 10.1089/10763270260424105

[CIT0018] Li X, Cui Q, Kao C, Wang GJ, Balian G. 2003 Lovastatin inhibits adipogenic and stimulates osteogenic differentiation by suppressing PPAR gamma 2 and increasing Cbfal/Runx 2 expression in bone marrow mesenchymal cell cultures. Bone. 33:652–659. 14555271 10.1016/s8756-3282(03)00239-4

[CIT0019] Meier C, Nguyen TV, Center JR, Seibel MJ, Eisman JA. 2005 Bone resorption and osteoporotic fractures in elderly men: the Dubbo Osteoporosis Epidemiology study. J Bone Miner Res. 20:579–587. 15765176 10.1359/JBMR.041207

[CIT0020] Maeda S, Hayashi M, Komiya S, Imamura T, Miyazono K. 2004 Endogenous TGF-beta signaling suppresses maturation of osteoblastic mesenchymal cells. EMBO J. 23:552–563. 14749725 10.1038/sj.emboj.7600067 PMC1271802

[CIT0021] Okazaki K, Sandell LJ. 2004 Extracellular matrix gene regulation. Clin Orthop. 427:S123–S128. 10.1097/01.blo.0000144478.51284.f3 15480054

[CIT0022] Na L, Min Z, Gregor P, Zhao Y, Tan YF, Luo S, Bo Qu X. 2016 Sika deer antler collagen type I-accelerated osteogenesis in bone marrow mesenchymal stem cells via the Smad pathway. Evid Based Complement Alternat Med. 2016:2109204. 27066099 10.1155/2016/2109204 PMC4809101

[CIT0023] Prophet E, Mills B, Arrington JB, Sobin L. 1994 Laboratory methods in histotechnology. Washington: Armed Forces Institute of Pathology.

[CIT0024] Sharp CA, Magnusson P. 2008 Isoforms of bone alkaline phosphatase, stem cells, and osteoblast phenotypes. Stem Cells Dev. 17:857–858. 18412517 10.1089/scd.2008.0085

[CIT0025] Szulc P, Montella A, Delmas PD. 2008 High bone turnover is associated with accelerated bone loss but not with increased fracture risk in men aged 50 and over: the prospective MINOS study. Ann Rheum Dis. 67:1249–1255. 18065499 10.1136/ard.2007.077941

[CIT0026] Sun P, Watanabe H, Takano K, Yokoyama T, Fujisawa J, Endo T. 2006 Sustained activation of M-Ras induced by nerve growth factor is essential for neuronal differentiation of PC12 cells. Genes Cells. 11:1097–1113. 16923128 10.1111/j.1365-2443.2006.01002.x

[CIT0027] Takeda S, Bonnamy JP, Owen MJ, Ducy P, Karsenty G. 2001 Continuous expression of Cbfα1 in nonhypertrophic chondrocytes uncovers its ability to induce hypertrophic chondrocyte differentiation and partially rescues Cbfα1 dificient mice. Genes Dev. 15:467–481. 11230154 10.1101/gad.845101 PMC312629

[CIT0028] Wenstrup RJ, Witte DP, Florer JB. 1996 Abnormal differentiation in MC3T3-E1 preosteoblasts expressing a dominant-negative type I collagen mutation. Connect Tissue Res. 35:249–257. 9084663 10.3109/03008209609029198

[CIT0029] Whitehurst A, Cobb MH, White MA. 2004 Stimulus-coupled spatial restriction of extracellular signal-regulated kinase 1/2 activity contributes to the specificity of signal-response pathwaysl. Mol Cell Biol. 24:10145–10150. 15542825 10.1128/MCB.24.23.10145-10150.2004 PMC529024

[CIT0030] Wu F, Li H, Jin L, Li X, Ma Y, You J, Li S, Xu Y. 2013 Deer antler base as a traditional Chinese medicine: a review of its traditional uses, chemistry and pharmacology. J Ethnopharmacol. 145:403–415. 23246455 10.1016/j.jep.2012.12.008

